# What causes human cancer? Approaches from the chemistry of DNA damage

**DOI:** 10.1186/s41021-016-0046-8

**Published:** 2016-07-01

**Authors:** Hiroshi Kasai

**Affiliations:** Department of Environmental Oncology, Institute of Industrial Ecological Sciences, University of Occupational and Environmental Health, 1-1, Iseigaoka, Kitakyushu, Yahatanishi-ku 807-8555 Japan

## Abstract

To prevent human cancers, environmental mutagens must be identified. A common mechanism of carcinogenesis is DNA damage, and thus it is quite possible that environmental mutagens can be trapped as adducts by DNA components. It is also important to identify new types of DNA damaging reactions and clarify their mechanisms. In this paper, I will provide typical examples of our efforts to identify DNA damage by environmental agents, from chemistry-based studies. **1) Oxidative DNA damage**: 8-Hydroxydeoxyguanosine (8-OHdG, 8-oxodG) was discovered during a structural study of DNA modifications caused *in vitro* by heating glucose, which was used as a model of cooked foods. We found that various oxygen radical-forming agents induced the formation of 8-OHdG in DNA, *in vitro* and *in vivo*. Analyses of the urinary 8-OHdG levels are useful to assess the extent of oxidative DNA damage in a human population. **2) Lipid peroxide-derived DNA adducts**: We searched for mutagens that react with deoxynucleosides, in model systems of lipid peroxidation. The reaction mixtures were analyzed by high performance liquid chromatography (HPLC), and we discovered various lipid peroxide-derived mutagens, including new mutagens. Some of these adducts were detected in human DNA. These mutagens may be involved in lipid peroxide-related cancers. **3) Methylation of cytosine by free radicals**: Methylation of the cytosine C-5 position is an important mechanism of carcinogenesis, in addition to gene mutations. However, the actual mechanisms of *de novo* methylation in relation to environmental agents are not clear. We found that cytosine C-5 methylation occurred by a free radical mechanism. The possible role of this radical-induced DNA methylation in carcinogenesis will be discussed, in relation to the presently accepted concept of cancer epigenetics. In these studies, chemical analyses of the adducts formed in model reactions led to the discoveries of new mutagens and important types of DNA modifications, which seem to be involved in human carcinogenesis.

## Background

**Fig. 1:**
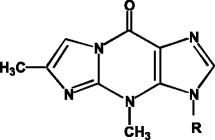
Structure of wyosine

In my early research as a graduate student (1969–1974) and a post-doctoral fellow (1975–1977) in natural product chemistry laboratories, I was involved in the determination of the structures of small amounts of naturally occurring modified nucleosides in transfer RNA (tRNA) and carcinogen-bound nucleosides. Among the modified nucleosides, an interesting discovery, published as my first research paper, was the fluorescent, methylated 1,N^2^-etheno-guanine-derivatized Yt base (wyosine nucleoside) in *T. utilis* tRNA^phe^ [1, 2] (Fig. 1), which is biosynthesized from 3-methylguanine. I was intrigued by a previous report, in which an ethenoguanine derivative was formed in DNA by the reaction of deoxyguanosine (dGuo) residues with the carcinogen glycidaldehyde [3, 4]. In tRNA, etheno-G stabilizes the tertiary structure of the anticodon-loop by stacking interactions with neighboring bases, leading to correct codon-anticodon interactions [5]. However, the etheno-G formed in DNA induces mutations by disturbing the DNA structure by stacking with neighboring bases, and by blocking normal G-C base-pairing by the 1,N^2^-etheno-modification [6]. After the study on the Yt base (wyosine nucleoside), my interest shifted to the modifications of nucleic acids by carcinogens. The Yt base actually “foreshadowed” the etheno-dG adducts formed by the hemin-ω-3-fat-peroxidation system, encountered in the latter part of my research period (2006).

As a postdoctoral fellow, I was involved in studies on modifications of nucleic acids by carcinogens, such as benzo[a]pyrene (BP) and 7,12-dimethylbenz[a]anthracene (DMBA), mostly in collaboration with a group at the Institute of Cancer Research, Columbia University [7–12]. These studies involved the isolation of modified nucleosides (BP-Guo, DMBA-Guo) on a microgram scale and their structure determination by mass-, UV-, CD-, and NMR-spectra. These micro-techniques in organic chemistry actually formed the basis for my subsequent chemical studies on DNA adducts. These experiences also gave me hints toward the idea that environmental mutagens can be trapped as nucleoside-mutagen-adducts.

On the occasion of the 10th anniversary of the foundation of the Journal, Genes and Environment, I am summarizing my 35 years of work on chemistry-based studies of DNA damage. This is also a review of my plenary lecture at ICEM Brazil in 2012, and my presentation at the Kitashi Mochizuki Memorial Symposium, Molecular Mechanisms of Mutagenesis, during the 2015 JEMS Meeting at Fukuoka, Japan.

## Introduction

**Fig. 2:**
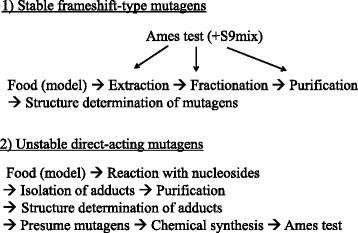
Methods to identify mutagens

**Fig. 3:**
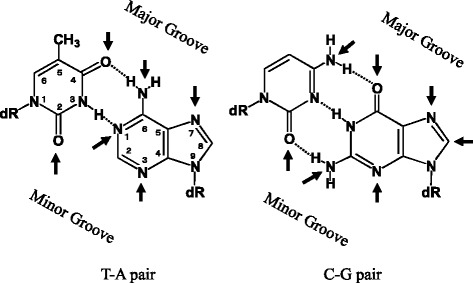
Mutagen reactive sites (indicated by arrows) in DNA. In addition, mutagens react with the oxygen atoms of phosphodiester bonds

Based on epidemiological studies, diet and smoking are the main causes of human cancer [13]. To prevent cancer, it is important to identify unknown mutagens and carcinogens, especially in food, because in regard to smoking it is best to stop the habit, regardless of the mutagens in cigarette smoke. To isolate mutagens in food, the extraction, fractionation, and final purification are usually conducted based on the mutagenic activity, using bacterial mutagenicity tests such as the Ames test (Fig. 2-1). This method is especially useful for isolating stable frameshift-type mutagens, monitored by the Ames test with S9 mix. This test is generally used by many researchers to isolate mutagens. Using this method, I was involved in the isolation of the heterocyclic amine mutagen, 2-amino-3-methylimidazo[4,5-*f*]quinoline (IQ), from broiled sardines, in collaboration with Drs. T. Sugimura and M. Nagao of the National Cancer Center Research Institute, Tokyo [14, 15]. However, the isolation of directly acting mutagens is difficult, because of their instability. A common mechanism of carcinogenesis is DNA damage [16], and many mutagens and carcinogens react with DNA, at the positions indicated by arrows (Fig. 3). Guanine (Gua) is the most reactive with mutagens. Thus, I thought it was quite possible that environmental mutagens, especially directly acting mutagens, could be trapped as Gua-adducts, and guanosine derivatives with a fluorescent group at the sugar moiety may be useful for mutagen-adduct analyses with high sensitivity, using HPLC coupled with a fluorescent detector (HPLC-Fl). For the structural characterization of adducts derived from unknown mutagens, certain amounts of the adducts (at least 300-500 micrograms) must be isolated for spectral measurements, such as mass-, UV-, and ^1^H- and ^13^C-NMR-spectra. Based on the determined structures of the adducts, the original mutagens can be presumed, and the mutagenicity can be confirmed using synthetic samples (Fig. 2-2).

In this paper, I discuss typical examples of our studies to identify DNA (nucleosides) damage induced by model systems, such as heated glucose, ω-3-fat-hemin-, and ω-6-fat hemin-peroxidation systems, especially in relation to diet. It is also important to identify new types of DNA damaging reactions and clarify their mechanisms. Accordingly, we examined the methylation of cytosine C-5 by methyl radicals, generated from environmental and endogenous compounds. This may be related to epigenetic changes during carcinogenesis, in relation to inflammation.

### Sensitive detection of mutagens as adducts with a fluorescent guanosine derivative

**Fig. 4:**
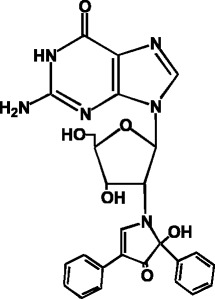
Structure of the fluorescent guanosine derivative, FG

**Fig. 5:**
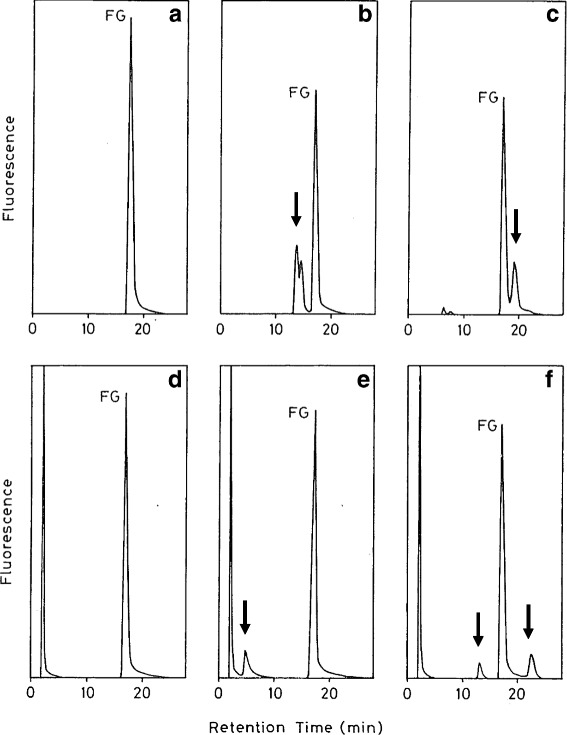
Analysis of standard mutagen-FG adducts by HPLC-Fl. **a** control FG, (**b**) glyoxal + FG, (**c**) methylglyoxal + FG, (**d**) control FG + S9 mix, (**e**) 4NQO + FG + S9 mix, (**f**) AF-2 + FG + S9 mix. Adduct peaks are indicated by arrows

**Fig. 6:**
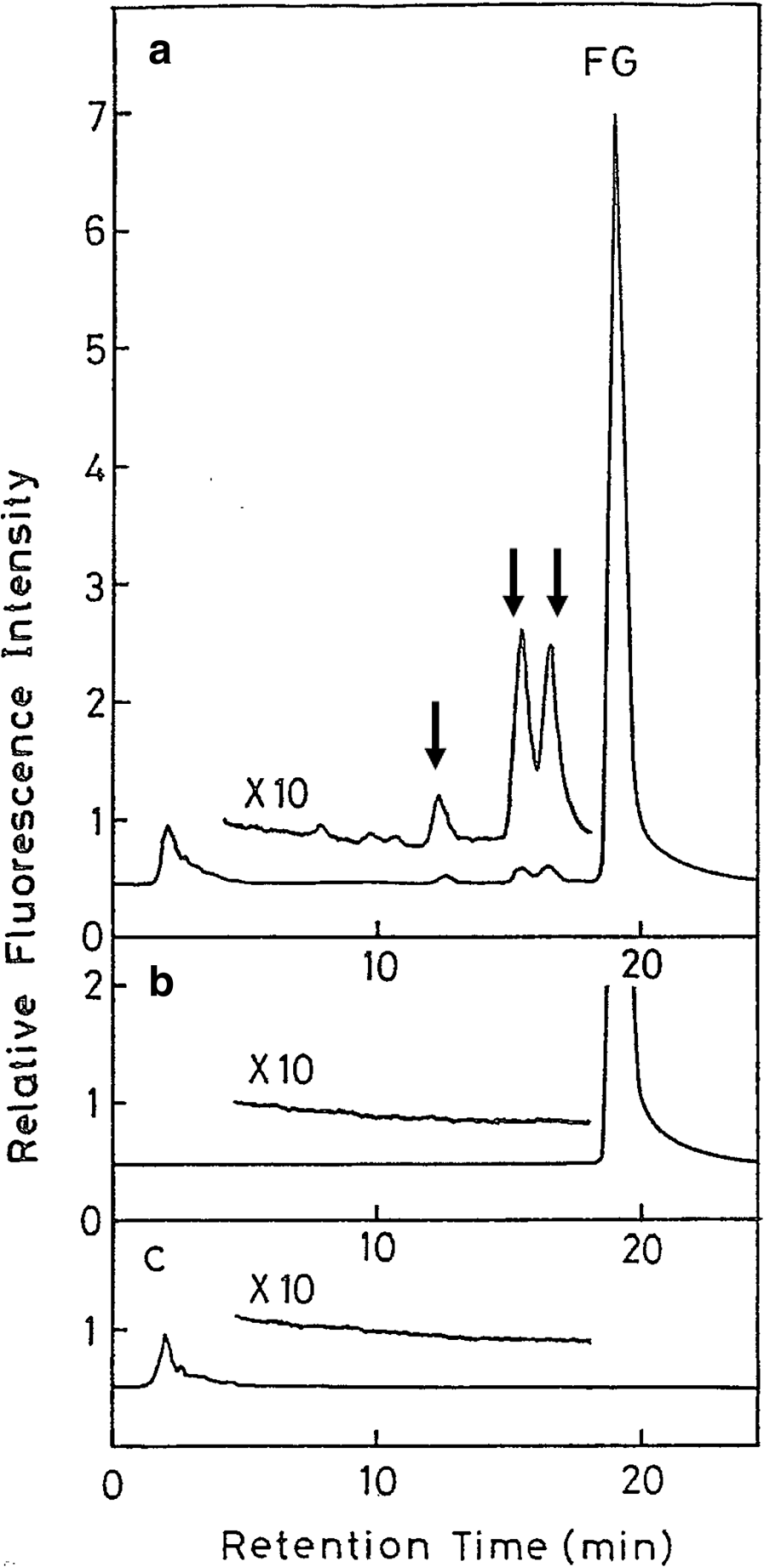
Analysis of mutagen-FG adducts formed by heated glucose. **a** FG + heated glucose, (**b**) control FG, (**c**) control heated glucose. Adduct peaks are indicated by arrows

As sensitive methods for analyses of carcinogen-bound DNA adducts, the ^32^P-postlabeling and LC/MS/MS methods are widely used. However these methods are complicated (^32^P-postlabeling) or require expensive equipment (LC/MS/MS), and thus are not suitable for general adduct analyses. To detect mutagen-guanosine adducts with high sensitivity, the highly fluorescent guanosine derivative, 2’-deoxy-(2”, 3”-dihydro-2”, 4”-diphenyl-2”-hydroxy-3”-oxo-1”-pyrrolyl)guanosine (FG) (Fig. 4), was prepared [17]. As the base moiety of FG is the same Gua as in DNA, it is expected that mutagens will react with FG as well as DNA. In fact, after reactions of FG and known mutagens, such as glyoxal, methylglyoxal, 4-nitroquinoline N-oxide (4NQO), and [2-(2-furyl)-3-(5-nitrofuryl)-acrylamide (AF-2) with or without S9mix, FG-mutagen-adducts were detected by HPLC-Fl analyses (Fig. 5). This method is also useful for the detection of adducts induced by unknown environmental mutagens in complex mixtures. For example, adduct formation by the reaction of FG and mutagenic heated glucose was revealed by the HPLC-Fl analysis method (Fig. 6) [18]. I hope that this method will be widely used in the future to test for adduct formation by various food extracts that may contain mutagens.

### Oxidative DNA damage: 8-Hydroxydeoxyguanosine

**Fig. 7:**
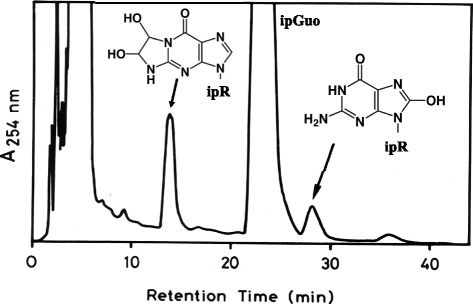
Identification of isopropylidene guanosine (ipGuo) adducts formed by mutagenic heated glucose

When glucose is heated at 200 °C for 20 minutes, as a model reaction of cooking, it becomes mutagenic to *S. typhimurium* TA100 in the absence of S-9 mix. It was difficult to isolate the mutagens themselves, because they are unstable and some of them are volatile. Therefore, we analyzed the adducts formed after the reaction of heated glucose and a guanosine derivative, isopropylidene guanosine (ipGuo). The reason for the use of ipGuo, instead of Guo (guanosine) or dGuo, is that ipGuo adducts can be extracted with organic solvents such as ethyl acetate, while most of the unimportant water-soluble materials remained in the aqueous reaction mixture, and thus the adducts can be efficiently analyzed by HPLC after the extraction. To detect mutagen-ipGuo adducts, it is important to compare the HPLC profiles of the heated glucose-ipGuo reaction mixture (Fig. 7), the control of heated glucose only, and the control of ipGuo only. Only the peaks in the HPLC-(reaction mixture), which are absent in the HPLC-(controls), are the real adducts formed between mutagens and ipGuo. We found two adducts, a glyoxal-ipGuo adduct and 8-OH-ipGuo. The latter adduct, 8-OH-ipGuo, was a new type of modification at that time, and thus we studied the mechanism of its formation.

**Fig. 8:**
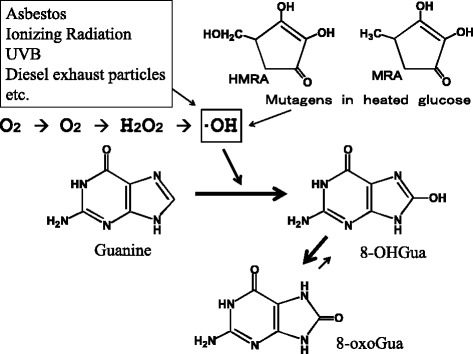
Formation of 8-OHGua by ROS

Soon after the detection of the 8-OH-modification of ipGuo, we found that the dG → 8-OHdG modification is induced in the nucleoside and DNA by reactive oxygen species (ROS), such as hydroxyl radicals ( • OH) (Fig. 8) [19, 20]. Asbestos, UVB, diesel exhaust particles, and ionizing radiation, etc. induced 8-OHdG in DNA via ROS production [21]. Actually, heated starch (polymer of glucose) contained two ROS-forming mutagens, methylreductic acid (MRA) and hydroxymethylreductic acid (HMRA), which are five-membered ring compounds [22]. Considerable amounts of these compounds were detected in various heat-processed foods, in the range of 17-904 microgram/g food. In addition to ROS, 8-OHdG was also produced by other mechanisms, such as the hydration of 7-aryl-dGuo [23], the hydration of a guanine radical cation [24], long-range electron transfer [25], etc.

**Fig. 9:**
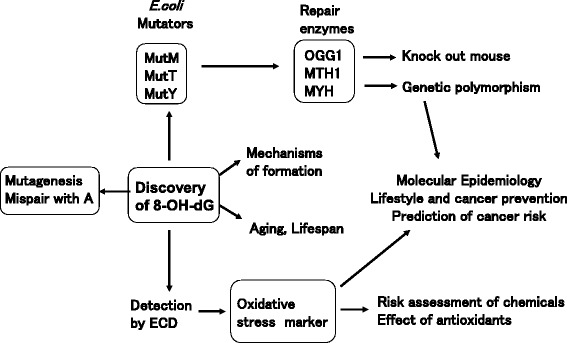
The discovery of 8-OHdG triggered important progress in many fields

Since the discovery of 8-OHdG formation in DNA, world-wide studies have been conducted on this modification from various aspects, such as mutation, repair and the mechanisms of its formation (Fig. 9). The biological significance of 8-OHdG was especially recognized after the detection of its mutagenic effects and the presence of its repair enzymes in mammalian cells [26]. It mispairs with A and induces the GC → TA transversion mutation [27]. Based on their homology to the *E. coli* mutator genes, *MutM, MutY*, and *MutT*, the 8-OHdG related repair enzymes OGG1, MYH, and MTH1 were identified in mammalian cells [28, 29], which led to studies on the knock-out mouse and genetic polymorphisms in the human population. After Floyd et al. reported that 8-OHdG can be detected with high sensitivity by an electrochemical detector (ECD) coupled to an HPLC [30], it was widely analyzed as a biomarker of oxidative stress, including studies on risk assessments of chemicals, effects of antioxidants, relationships to various ROS-related diseases, and aging.

**Fig. 10:**
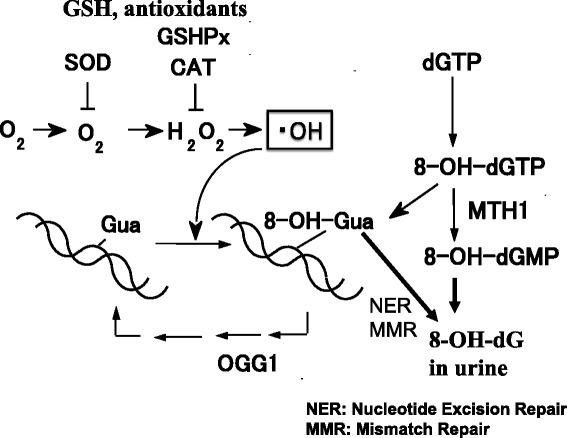
Sources of urinary 8-OHdG

Over the past two decades, we have made efforts to establish an accurate method to measure 8-OHdG in cellular DNA and urine, as a biomarker of cellular oxidative stress in animals and humans [21, 31, 32]. I developed a simple and accurate method for urinary 8-OHdG analysis by HPLC-ECD [33], because the widely used ELISA method is inaccurate, due to the cross-reaction of the antibody with urea, a major urinary component [34]. The possible sources of urinary 8-OHdG may be the hydrolysis of 8-OHdGTP by the sanitization enzyme MTH1, nucleotide excision repair, or mismatch repair (Fig. 10) [32, 35]. The free base 8-hydroxyguanine (8-OHGua) is also a good marker of oxidative stress [36, 37]. For the analysis of urinary 8-OHGua, the diet must be carefully controlled. For instance, the standard rodent diet CE-2 (CLEA) in animal experiments and fish consumption in human studies should be avoided [38], because these food sources contain large amounts of 8-OHGua, and 90 % of it is excreted into the urine within one week [31]. For animal experiments, we use a diet containing egg white as the protein source, which contains 50-fold less 8-OHGua as compared to CE-2. As sources of urinary 8-OHGua, base excision repair from oxidized DNA and the oxidation of Gua released by the hydrolytic degradation of DNA, RNA and nucleotides are possible [32].

The number of published reports with 8-OHdG in the title is presently 1,420, and most have focused on human urine. The reports include the effects of chemicals (animal, 70; human, 120), radiation (84), diseases (494), lifestyles (694) and antioxidants (229). For example, in relation to dietary habits, when a vitamin-deficient diet (for two months) and a commercially available sweet beverage (for two weeks) were administered to mice, the urinary 8-OHdG levels clearly increased [39]. These results indicated that oxidative stress could be elevated by the prolonged intake of an unbalanced diet. Regarding the risk of ionizing radiation, the relationship between low dose (<100 mSv) radiation and cancer risk is still unclear. We studied the effects of low dose radiation on 8-OHdG formation in mouse DNA and urine [40]. After whole body irradiation by X-rays, the 8-OHdG levels in liver DNA and urine increased from the 500 and 200 mGy doses, respectively. These results indicated that living organisms have a defense system against ionizing radiation, and a threshold seems to exist for oxidative DNA damage. Regarding human occupational and environmental exposure to chemicals, increases of 8-OHdG were detected in relation to exposure to benzene, ethylbenzene, styrene, trichloroethylene, polycyclic aromatic hydrocarbons, di-(2-ethylhexyl)phthalate (plastic recycling), PCBs, dioxin, As, Cr, Cd, Ni, Se, nanoparticles (copier), PM2.5 in diesel exhaust particles, and environmental tobacco smoke. In addition, elevated 8-OHdG levels were detected in workers in the asbestos-, azo-dye-, and rubber-industries, coke oven workers, foundry workers, bus drivers, traffic policemen, hair salon employees (volatile organic compounds), people exposed to cooking oil fumes, ash treatment, and an antineoplastic drug (5-FU), and agricultural workers (organophosphate). For more information, please see the review papers [21, 41, 42].

### Lipid peroxide-derived DNA adducts

**Fig. 11:**
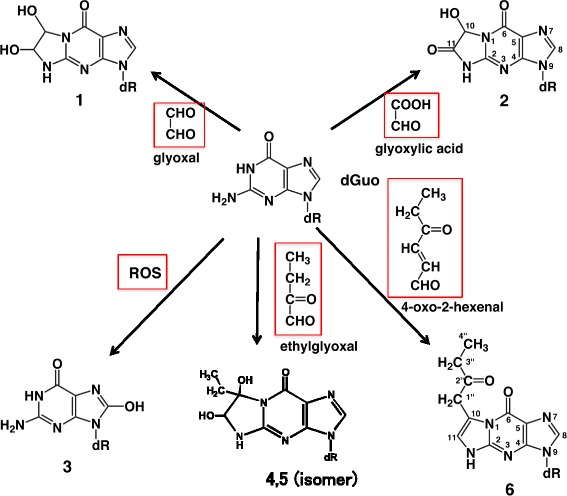
Adduct formation of dGuo in an ω-3-fat-hemin peroxidation model system. Mutagens involved in the adduct formation are shown in the red boxes

A high fat diet is a risk factor for various cancers, such as breast and prostate cancer [43, 44], and an elevated risk of colon cancer is associated with red meat consumption [45]. Based on epidemiological studies, a higher intake of heme iron is positively associated with colorectal cancer risk [46]. The simultaneous feeding of a high fat diet and heme iron significantly increased the incidence of colon cancer in rats [47]. Therefore, it is interesting to study adduct formation by a lipid peroxidation model system, containing unsaturated fatty acid and hemin. First, I will mention the results of ω-3-fat peroxidation. When dGuo was reacted with a model system of an emulsion of linoleic acid plus hemin, 6 adduct peaks were detected by HPLC. The structures of the possible mutagens involved in the adduct formation are shown in Fig. 11 [48]. Adduct 1 is produced by glyoxal, adduct 2 is produced by glyoxylic acid, adduct 3, 8-OHdG, is produced by ROS, and adducts 4 and 5 are produced by ethyl glyoxal. Adduct 6 showed a unique UV-spectrum. I noticed that it is very similar to that of wyosine, described in the Preface. It showed strong fluorescence on a TLC plate, similar to wyosine. The structure was determined to be 1,N^2^-etheno-dGuo with a 2-oxobutyl side chain (structure 6). It was proposed to be formed by the reaction of 4-oxo-2-hexenal (4-OHE) and dGuo. The synthetic 4-OHE showed mutagenic activity in the TA100 and TA104 strains without S9 mix. Among these possible mutagens, 4-OHE and ethyl glyoxal have not been reported thus far. In addition to dGuo, 4-OHE also reacts with dCyd (deoxycytidine), m^5^dCyd (5-methyldeoxycytidine) and dAdo (deoxyadenosine) to give etheno-type adducts, *in vitro*. After the oral administration of 4-OHE to mice, these adducts were found in the mouse stomach, as detected by the LC/MS/MS method [49]. The detection range was 1-2 adducts per 10^5^ nucleosides. These adducts were also detected in human tissues, including the stomach, by the LC/MS/MS method, as reported by Matsuda and his collaborators [50]. We also determined the amounts of 4-OHE in cooked foods [51]. Considerably large amounts of 4-OHE were detected in cooking oil and broiled fish, in the range of 10 micrograms per g food.

**Fig. 12:**
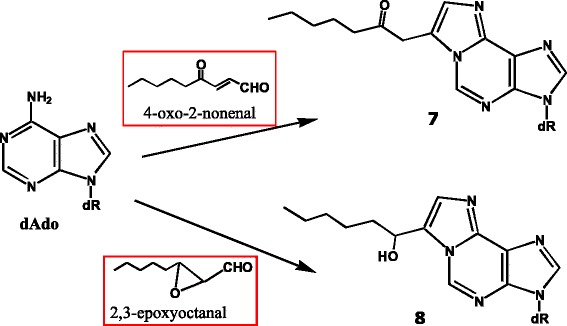
Adduct formation of dAdo in an ω-6-fat-hemin peroxidation model system. Mutagens involved in the adduct formation are shown in the red boxes

**Fig. 13:**
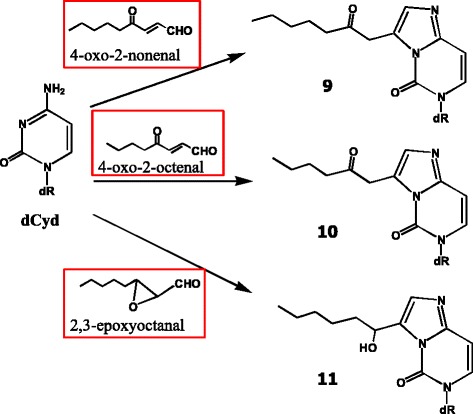
Adduct formation of dCyd in an ω-6-fat-hemin peroxidation model system. Mutagens involved in the adduct formation are shown in the red boxes

Next, we examined adduct formation between nucleosides and an ω-6 fat peroxidation model system [52]. Based on the literature, ω-6 fat, rather than ω-3-fat, and red meat seem to be more important risk factors in relation to colon cancer [53]. For this purpose, a model system, hemin plus ethyl linoleate, was reacted with deoxynucleosides in an emulsion, and the reaction products were analyzed by HPLC. As a result, etheno-type adducts were detected in the reaction mixtures with dAdo (Fig. 12, structures 7 and 8) and dCyd (Fig. 13, structures 9, 10, and 11). With dGuo, the adduct yield was low.

The mutagen involved in the formation of adducts 8 and 11 seemed to be 2,3-epoxyoctanal. In fact, synthetic 2,3-epoxyoctanal showed potent mutagenicity in the TA 100 and TA104 strains without S-9 mix. As many epoxy compounds are carcinogenic, it is quite possible that this compound may be involved in human carcinogenesis. It can be formed by various pathways; for example, from 9-hydroperoxy-octadecadienoic acid via 2,4-decadienal and 2-octenal, or from 13-hydroperoxy-octadecadienoic acid via an epoxy derivative. We found that 2,3-epoxyoctanal readily formed under acidic conditions. These results suggested that this mutagen could be efficiently formed during storage and cooking, or during digestion in the stomach, under acidic conditions.

A known mutagen, 4-oxo-2-nonenal (4-ONE), was presumed to be involved in the formation of adducts 7 and 9. In addition, 4-oxo-2-octenal (4-OOE) was proposed as the possible mutagen involved in the formation of the dCyd adduct 10.

Notably, the adducts formed between nucleosides and lipid peroxide-derived mutagens (4-OHE, 4-ONE, 4-OOE, 2,3-epoxyoctanal) were all detected in human tissue DNA [50, 54].

### Methylation of cytosine by free radicals

In addition to genetic changes, epigenetic changes are an important mechanism of aberrant gene expression and carcinogenesis. Environmental factors and dietary and lifestyle factors are closely related to the induction of both genetic and epigenetic changes. A key molecule involved in epigenetic change is 5-methyl-2’-deoxycytidine (m^5^dCyd). Methylation of CpG islands is associated with gene silencing, while DNA hypermethylation of tumor suppressor genes plays a critical role in carcinogenesis [55]. It is widely accepted that the methyl group is enzymatically introduced at the dCyd C-5 position. DNA methyltransferases are involved in both *de novo* methylation and maintenance methylation [56]. After DNA methylation, methylated DNA binding domain protein (MBD) binds to the methylated site, a histone deacetylase is recruited, and finally gene inactivation occurs [57]. However, the exact mechanisms of hypermethylation, particularly in relation to environmental factors, are not clear.

**Fig. 14:**
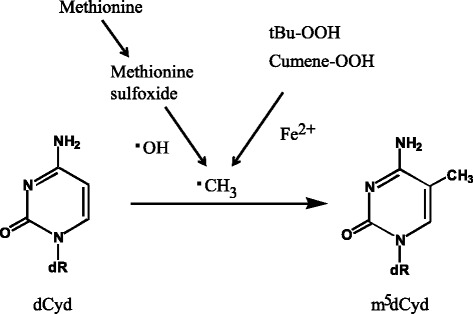
Free radical-mediated dCyd C-5 methylation

It is interesting to propose a free radical mechanism for the formation of m^5^dCyd, because the C-8-methylation of dGuo by a methyl radical has been reported [58, 59]. In these studies, carcinogens, methyl hydrazine and dimethylhydrazine, and tumor promoters, t-Bu-OOH (t-butyl hydroperoxide) and cumene-OOH, were used to generate methyl radicals in the presence of Fe (iron). In our study, we tested the formation of m^5^dCyd from dCyd by Bu-OOH and cumene-OOH. When dCyd and Cu-OOH were reacted in the presence of ferrous ion, m^5^dCyd formation was clearly detected (Fig. 14) [60]. The retention time and the UV spectrum of the reaction product were the same as those of the standard m^5^dCyd. The formation of m^5^dCyd was also confirmed by an immune-dot blot analysis. In these experiments, we demonstrated the formation of m^5^dCyd by methyl radicals, produced from the side chains of t-Bu-OOH and cumene-OOH. Cumen hydroperoxide and t-butyl hydroperoxide are environmental chemicals [61]. Large amounts of these chemicals are used as raw materials for the production of acetone and phenol, as intermediates for organic synthesis, and as catalysts for polymerization.

As another example, I will summarize our results about dCyd methylation by methionine sulfoxide (MetO) plus • OH [62]. Methionine sulfoxide is an oxidized product of methionine, and a biomarker of oxidative stress. It is generated in proteins by smoking [63], inflammation [64] and aging [65]. We confirmed that MetO generated methyl radicals with an oxygen radical forming system, and modified dCyd to m^5^dCyd, *in vitro* (Fig. 14). To confirm whether this reaction is biologically relevant, MetO was administered to non-alcoholic steatohepatitis (NASH) mice [66]. It is well known that NASH mice have high oxidative stress in the liver. The incidences of hepatocellular carcinoma were higher in the MetO-administered groups. The multiplicity (number of tumors per mouse) was also increased in the MetO-administered groups. We also analyzed DNA methylation, by methylation-specific PCR. The DNA methylation status of the p16 gene promoter region was higher in the livers of the MetO-treated mice. These results suggested that MetO plus ROS actually triggers DNA methylation via methyl radicals *in vivo*.

**Fig. 15:**
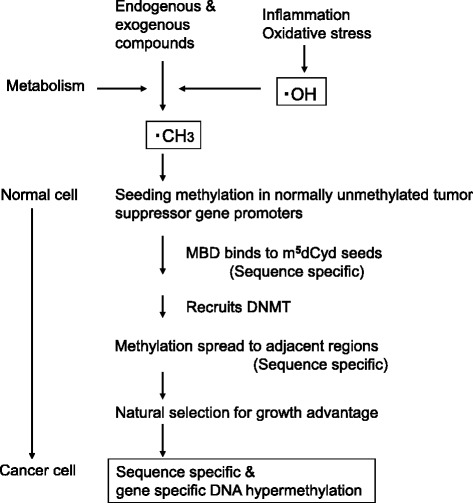
Possible roles of free radicals in epigenetic changes during carcinogenesis

A general scheme of methyl radical formation and dCyd methylation under oxidative stress conditions is shown in Fig. 15 [67, 68]. Endogenous compounds such as MetO, induced by smoking, inflammation and aging, can generate methyl radicals under oxidative stress conditions. Exogenous compounds, such as t-Bu-OOH, cumene-OOH, dimethylhydrazine, and acetaldehyde, reportedly generate methyl radicals. It is possible that cytosine methylation occurs randomly in DNA. Clark and her collaborators proposed that a low level of seeding methylation occurs randomly at a very early stage of epigenetic change [69]. In the latter steps, sequence- and gene-specific DNA methylation occurs by MBD binding and the recruitment of DNMT (DNA methyltransferase), leading to a cancer-specific methylation pattern. We think our discovery of free radical-mediated cytosine methylation is related to this seeding methylation. Further studies are needed to assess the possibility of this radical DNA methylation mechanism in cellular DNA.

## Discussion

About 90–95 % of cancers are induced by environmental factors, including diet (30–35 %) and smoking (25–30 %) [13]. Although the detailed mechanisms of carcinogenesis, such as gene mutations and epigenetic changes, are being clarified by recent progress in the molecular biology of cancer, the global identification of mutagens, especially in food, is necessary for cancer prevention. Only a limited number of food mutagens, such as aflatoxin B1, *N*-nitrosamines, polycyclic aromatic hydrocarbons, heterocyclic amines, have been identified in foods [70]. I think they are only the tip of the iceberg, if we consider the thousands of unknown food mutagens. Methods such as ^32^P-postlabeling and LC/MS/MS can be used to detect many DNA adducts in human DNA with high sensitivity; however, the detected adducts are mostly unknown and none of the above known mutagens are the major sources of the adducts [71, 72]. We are currently only able to assess human cancer risk by using information such as the potency of genotoxicity, the amounts in foods, and the DNA adduct levels of these known mutagens. Without exhaustive research on food mutagens including unknown mutagens, risk assessments of food-derived cancer are either unreliable or impossible. To my great regret, fundamental studies to search for new environmental mutagens (not analyses of known mutagens), in food, air, and water, have seemed to decline recently. Young researchers are not interested in these projects, and have shifted their focus to molecular biology, because the former are high-risk; i.e., these projects require extensive efforts with the potential for meager outcomes. Looking back on my 35 years of research, fewer than 10 new discoveries of mutagens or DNA modifications were reported. They are the oxidative DNA damage 8-OHdG; the new mutagens MRA, HMRA, ethyl glyoxal, 4-oxo-2-hexenal, and 2,3-epoxyoctanal; and the methylation of cytosine C-5 by a free radical mechanism, etc. These studies were neither efficient nor elegant, because the daily experiments were mostly fruitless and interesting results were only obtained on very rare occasions. Fortunately, most of the adducts detected by HPLC (7 out of 11) were new types of modifications, except for the adducts with ONE, glyoxal, and glyoxylic acid. Furthermore, many of the adducts were detected in human DNA by LC/MS/MS. Especially, 8-OHdG was the seed for many important research developments in various fields, as shown in Fig. 9. In a sense, these unique chemistry-based approaches are more efficient for new discoveries, as compared to molecular biology-based approaches. In the latter approaches, many researchers must compete to obtain new findings with similar projects in the modern trend.

Further chemistry-based studies to search for new environmental mutagens and DNA modifications should be encouraged, especially for young researchers.

## Conclusion

To prevent human cancers, environmental mutagens must be identified. Chemical analyses of the adducts formed in model reactions led to the discoveries of new mutagens and important types of DNA modifications, which seem to be involved in human carcinogenesis.
